# Effects of Local Segregation on Stacking Fault Energy, Hydrogen Diffusion and Dislocation Motion in Austenitic Stainless Steel: A Molecular Dynamics Study

**DOI:** 10.3390/ma19101950

**Published:** 2026-05-09

**Authors:** Kaiyu Zhang, Wanliang Zhang, Chengshuang Zhou, Lin Zhang

**Affiliations:** Institute of Material Forming and Control Engineering, Zhejiang University of Technology, Hangzhou 310014, China; 2111825057@zjut.edu.cn (K.Z.); zhwl0525@gmail.com (W.Z.); zhoucs@zjut.edu.cn (C.Z.)

**Keywords:** austenitic stainless steel, local segregation, stacking fault energy, hydrogen diffusion, dislocation motion

## Abstract

Local chemical heterogeneity is a typical feature of selective laser melted (SLM) austenitic stainless steel and is closely related to its hydrogen-assisted deformation behavior. In this work, molecular dynamics simulations are performed to investigate the effects of local segregation on stacking fault energy, hydrogen diffusion, and dislocation motion in austenitic stainless steel. Three representative alloy compositions, Fe_71_Cr_17_Ni_12_, Fe_71_Cr_23_Ni_6_, and Fe_71_Cr_11_Ni_18_, are used to describe local composition variation associated with segregation in SLM-relevant austenitic stainless steel. The results show that Ni-rich regions exhibit relatively higher stacking fault energy and faster hydrogen diffusion, whereas Cr-rich regions show lower stacking fault energy and reduced hydrogen mobility. Hydrogen further decreases the stacking fault energy in all three alloy models and exerts a stronger influence on local defect energetics than composition variation alone. Shear simulations indicate that elemental segregation itself has only a limited direct effect on dislocation motion, whereas its interaction with hydrogen leads to a more evident retardation of partial dislocation propagation within the segregation region. These findings highlight the coupled roles of local composition variation and hydrogen in governing defect evolution and local deformation behavior in segregation-containing regions.

## 1. Introduction

The global transition toward a sustainable hydrogen economy has placed immense focus on the structural integrity of materials used in high-pressure storage and cryogenic transport systems. Austenitic stainless steels are considered primary candidates for these infrastructures due to their exceptional low-temperature toughness and perceived hydrogen compatibility. However, hydrogen embrittlement (HE) remains a critical safety bottleneck, as the interaction between hydrogen and microstructural defects can lead to catastrophic failure. Selective laser melting (SLM) has recently emerged as a transformative additive manufacturing technique, enabling the production of these steels with unique hierarchical microstructures and superior mechanical properties. Unlike conventionally processed alloys, SLM-fabricated steels commonly exhibit a “signature” chemical heterogeneity, characterized by cellular or dendritic substructures accompanied by localized solute redistribution [1,2,3].

Experimental investigations into SLM-processed 316L have confirmed that rapid solidification produces Cr- and Mo-rich cellular walls together with significant compositional gradients between cell interiors and boundaries [4,5,6,7]. While advanced data-driven approaches, such as interpretable machine learning, have begun to provide macroscopic predictive frameworks for these multi-component systems [8], they often lack the resolution to decipher the underlying atomistic mechanisms. To establish a robust “material-by-design” strategy for hydrogen-resistant additive manufacturing, it is indispensable to gain fundamental insights into how this SLM-specific local chemistry alters specific defect behaviors.

The mechanical response of austenitic steels is fundamentally governed by the stacking fault energy (SFE), which dictates dislocation dissociation, planar slip, and the stability of the austenitic matrix [9,10,11,12]. In the context of SLM, local chemical heterogeneity is expected to influence hydrogen transport by modifying the migration pathways and local occupancy of interstitial H atoms [13]. These two issues-defect energetics and hydrogen kinetics—are inextricably linked in SLM materials because segregation develops concurrently with the complex defect structures during rapid solidification [14,15,16].

In hydrogen-containing environments, the coupling among local composition, hydrogen diffusion, and dislocation motion becomes the decisive factor in local deformation and eventual degradation. However, capturing the transient interactions between hydrogen and defects within nanoscale segregation zones remains a significant experimental challenge. Molecular dynamics (MD) simulations, therefore, provide a necessary bridge to clarify whether local segregation acts as a benign microstructural feature or a detrimental “weak link” under hydrogen service. By constructing representative local-composition models that capture the essential contrast between Cr-rich and Ni-rich regions [17,18], the present study aims to elucidate the atomistic mechanisms by which local chemistry alters hydrogen-related defect evolution in SLM-relevant austenitic stainless steels.

## 2. Materials and Methods

All molecular dynamics simulations were performed using the Large-scale Atomic/Molecular Massively Parallel Simulator (LAMMPS, 22 July 2025), and the atomic configurations were post-processed using OVITO (3.13.0) [19,20]. Dislocation extraction analysis (DXA) was employed to visualize the evolution of dislocations and their interaction with the segregation region, while common neighbor analysis (CNA) was used to identify local structural changes and stacking-faulted regions. The interatomic interactions among Fe, Cr, Ni, and H atoms were described by an EAM-type potential developed by Zhou et al. [21], and this potential accurately reproduces the stacking fault energy values and hydrogen-lattice interaction energies. It should be noted that the present study focuses on relative trends and atomistic mechanisms associated with local segregation and hydrogen, rather than absolute quantitative prediction of all material properties.

**Figure 1:**
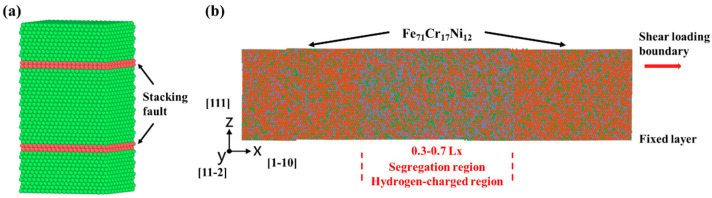
Schematic diagrams of the models used for stacking fault energy calculation (**a**) and shear simulation (**b**).

The effect of local composition variation on the stacking fault energy was investigated for three representative austenitic compositions, namely Fe_71_Cr_17_Ni_12_, Fe_71_Cr_23_Ni_6_, and Fe_71_Cr_11_Ni_18_. The selected compositions are designed to capture the chemical contrast between the matrix and segregation-affected regions (e.g., Cr-rich cellular walls and Ni-rich cell interiors) typically found in SLM-relevant microstructures. Random solid-solution models were constructed for each composition with dimensions of $50.8\;\times \;44.0\;\times \;93.3\;{Å}^{3}$ and containing 18,000 atoms per configuration. Hydrogen concentrations of 0, 0.5, and 1.0 at.% were introduced into interstitial sites. After energy minimization, an intrinsic stacking fault was introduced on a (111) plane by applying a rigid displacement corresponding to a Shockley partial Burgers vector, $\frac{a}{6}$<112>, to part of the crystal, as shown in Figure 1a. The SFE was calculated from the energy difference between the faulted and perfect configurations divided by the corresponding fault area. To reduce the influence of local chemical disorder, 2000 independent random configurations were generated for each hydrogen concentration, and the averaged value was taken as the SFE.

Hydrogen diffusion was studied for the same three compositions in order to clarify the influence of segregation-related local chemistry on hydrogen mobility. For each composition, periodic solid-solution models with dimensions of $92.7\;\times \;92.7\;\times \;92.7\;{Å}^{3}$ and containing 62,500 atoms were constructed. Hydrogen was introduced at a concentration of 1 at.%, corresponding to 625 H atoms in each simulation cell. The models were simulated at 950, 1050, and 1150 K under three-dimensional periodic boundary conditions. The temperature range of 950–1150 K was selected to accelerate hydrogen hopping events, ensuring sufficient statistical sampling for the calculation of diffusion coefficients within the nanosecond timescale of molecular dynamics simulations. After equilibration in the NPT ensemble, the mean square displacement (MSD) of hydrogen atoms was calculated, and the diffusion coefficient was obtained from the linear regime of the MSD-time relation according to the Einstein relation [14].

To investigate the influence of segregation on dislocation motion, a shear deformation model was established based on the Fe_71_Cr_17_Ni_12_ matrix, as shown in Figure 1b. The crystal orientation was set as <1-10>, <11-2>, and <111> along the three simulation axes, respectively, to facilitate the observation of partial dislocation glide on (111) planes. The simulation cell had dimensions of $382.0\;\times \;87.9\;\times \;99.5\;{Å}^{3}$ and contained 288,960 atoms. A segregation band was introduced in the central part of the simulation box, covering 30–70% along the x-direction. Within this region, random substitutional reassignment was used to change the local composition from Fe_71_Cr_17_Ni_12_ to approximately Fe_71_Cr_23_Ni_6_, while the surrounding matrix retained the baseline composition. In selected cases, 5 at.% H was further introduced into the segregation region to examine the combined effect of segregation and hydrogen. It should be noted that a hydrogen concentration of 5 at.% was introduced locally within the segregation region to represent the high local hydrogen occupancy often found at microstructural traps and to ensure sufficient sampling of hydrogen-dislocation interactions within the MD time and space scales.

Before loading, the models were minimized and equilibrated at 300 K. Shear deformation was then applied by imposing a constant velocity on the upper rigid layer, while the bottom layer was fixed and the middle atoms were free to respond. The corresponding engineering shear strain rate was $1.0\times 10^{-4}\;{ps}^{-1}$. The shear stress–strain response was recorded during deformation, and the atomic-scale evolution of dislocations was analyzed by OVITO.

## 3. Results

### 3.1. Stacking Fault Energy in Different Segregation States

**Figure 2:**
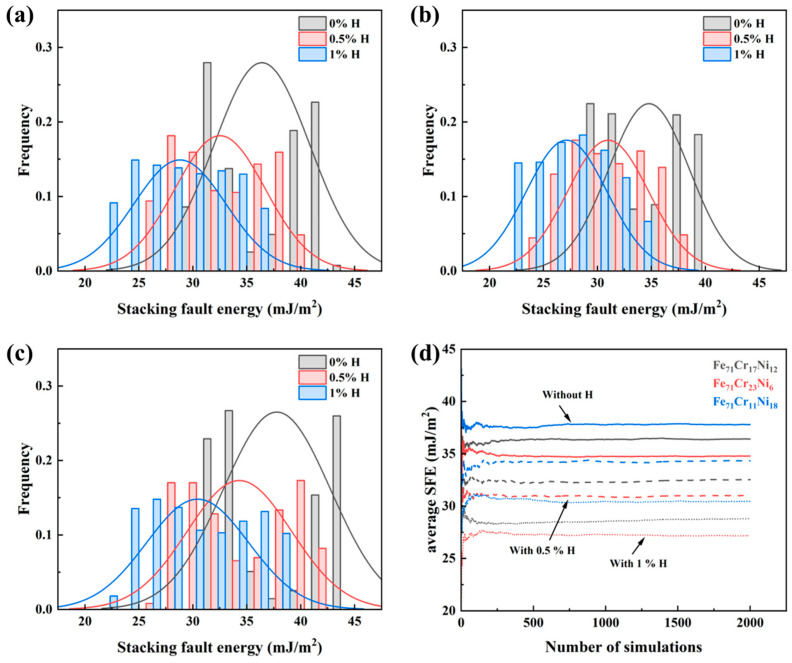
Statistical distributions and cumulative average values of stacking fault energy for three alloy compositions at different hydrogen concentrations: (**a**) Fe_71_Cr_17_Ni_12_; (**b**) Fe_71_Cr_23_Ni_6_; (**c**) Fe_71_Cr_11_Ni_18_; and (**d**) cumulative average SFE as a function of the number of simulations.

Figure 2 shows the statistical distributions and cumulative average values of the stacking fault energy for the three alloy compositions under different hydrogen concentrations. As shown in Figure 2a–c, all three alloy models exhibit distributed rather than single-valued SFE results, indicating that the local atomic arrangement in the random solid solution leads to noticeable statistical variation in the fault energy. Despite this fluctuation, systematic trends associated with both composition and hydrogen concentration can still be identified.

For all three compositions, the introduction of hydrogen shifts the SFE distribution toward lower values. Compared with the hydrogen-free condition, the distributions at 0.5% H and 1% H are generally displaced to the left, indicating a progressive decrease in SFE with increasing hydrogen concentration. This tendency is consistently observed in Fe_71_Cr_17_Ni_12_, Fe_71_Cr_23_Ni_6_, and Fe_71_Cr_11_Ni_18_.

A comparison among the three alloy compositions further shows that local chemistry has a clear effect on SFE. As summarized by the cumulative average curves in Figure 2d, Fe_71_Cr_11_Ni_18_ exhibits the highest average SFE under all hydrogen concentrations, whereas Fe_71_Cr_23_Ni_6_ shows the lowest values, with Fe_71_Cr_17_Ni_12_ lying in between. Therefore, the relative ranking of the three compositions remains unchanged over the investigated hydrogen concentration range.

Figure 2d also shows that the cumulative average SFE gradually converges with increasing number of simulations. Although fluctuations are present at the early stage of sampling, all curves become stable as the number of sampled configurations increases, indicating that the averaged SFE values are statistically reliable. To validate the quantitative accuracy of the present simulations, the calculated average SFE for the baseline Fe_71_Cr_17_Ni_12_ alloy ($\sim 36.4\;mJ/m^{2}$) is compared with established experimental and first-principles (DFT) results for austenitic stainless steels [9,22]. Our result falls well within the reported literature range for similar alloy systems, and shows good consistency with the theoretical predictions derived from the Fe-Cr-Ni-H interatomic potential used in this study. This quantitative agreement confirms that the random solid-solution models and the chosen interatomic potential accurately capture the fundamental defect energetics, providing a reliable basis for investigating the subsequent influence of hydrogen and local segregation.

### 3.2. Hydrogen Diffusion in Different Segregation States

**Figure 3:**
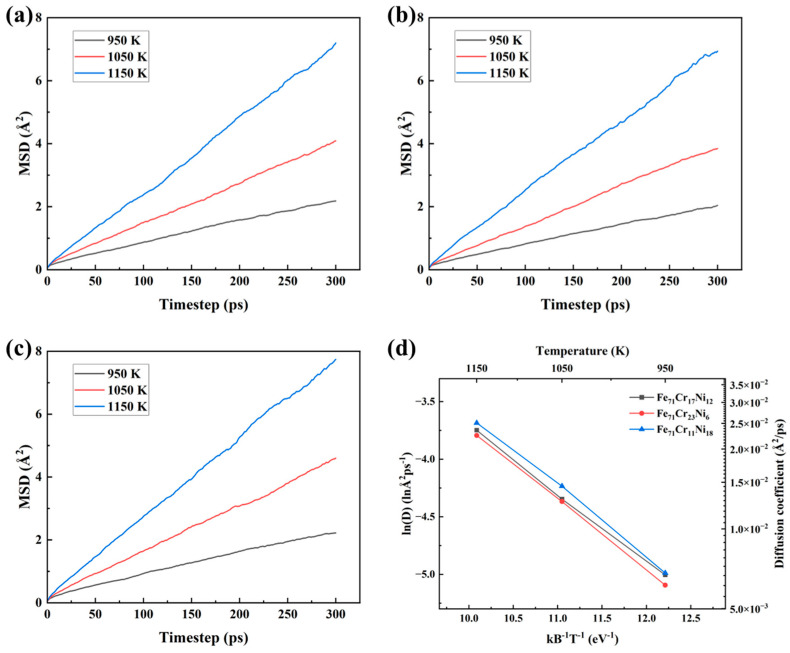
Hydrogen diffusion behavior in three alloy compositions at different temperatures: (**a**) MSD curves of Fe_71_Cr_17_Ni_12_; (**b**) MSD curves of Fe_71_Cr_23_Ni_6_; (**c**) MSD curves of Fe_71_Cr_11_Ni_18_; and (**d**) relationship between lnD and ${\left(kBT\right)}^{-1}$ derived from the MSD results.

Figure 3 shows the mean square displacement (MSD) of hydrogen atoms in the three alloy models at different temperatures, together with the corresponding relationship between lnD and ${\left(kBT\right)}^{-1}$. As shown in Figure 3a–c, the MSD increases approximately linearly with simulation time for all three compositions at 950, 1050, and 1150 K, indicating stable diffusive behavior of hydrogen within the present simulation timescale.

In all cases, the slope of the MSD curve increases with increasing temperature, showing that hydrogen mobility is enhanced at elevated temperatures [14]. A comparison among the three alloy compositions also reveals a clear compositional dependence. At the same temperature, the Fe_71_Cr_11_Ni_18_ model generally exhibits the largest MSD, whereas Fe_71_Cr_23_Ni_6_ shows the smallest MSD, with Fe_71_Cr_17_Ni_12_ lying in between. Since the diffusion coefficient is directly related to the slope of the MSD curve, this result indicates that hydrogen diffuses faster in the Ni-rich alloy and more slowly in the Cr-rich alloy.

The diffusion coefficients calculated from the MSD results are summarized in Figure 3d in the form of lnD versus ${\left(kBT\right)}^{-1}$. For all three compositions, lnD decreases nearly linearly with increasing ${\left(kBT\right)}^{-1}$, indicating that hydrogen diffusion follows an Arrhenius-type temperature dependence within the studied temperature range. The approximately parallel trends of the three fitted lines suggest that the temperature sensitivity of hydrogen diffusion is broadly similar for the different alloy compositions, whereas the absolute diffusion level is shifted by local composition. It should be noted that while the simulation temperatures are higher than typical service conditions, the strong Arrhenius linearity observed in our results suggests that the underlying diffusion mechanism remains consistent. Given that the activation energy for hydrogen diffusion in FCC lattices is relatively stable across a wide temperature range, the relative differences in hydrogen mobility observed between Ni-rich and Cr-rich regions are expected to persist at lower temperatures, albeit with lower absolute diffusion magnitudes.

Among the three models, Fe_71_Cr_11_Ni_18_ consistently exhibits the highest diffusion coefficient over the investigated temperature range, whereas Fe_71_Cr_23_Ni_6_ shows the lowest value. Overall, the results demonstrate that hydrogen diffusion in the present austenitic stainless steel models is affected by both temperature and local composition, with increasing temperature accelerating hydrogen mobility and Ni-rich/Cr-rich local chemistry corresponding to higher/lower diffusion rates, respectively.

### 3.3. Coupled Effects of Segregation and Hydrogen on Dislocation Motion

In FCC metals, a perfect dislocation usually dissociates into two Shockley partial dislocations separated by a stacking-fault ribbon on the (111) slip plane. In the present work, this defect structure was identified by CNA, and only the stacking-fault atoms were retained for visualization. Therefore, the position and width of the stacking-fault ribbon were used to characterize the motion of the dissociated dislocation.

**Figure 4:**
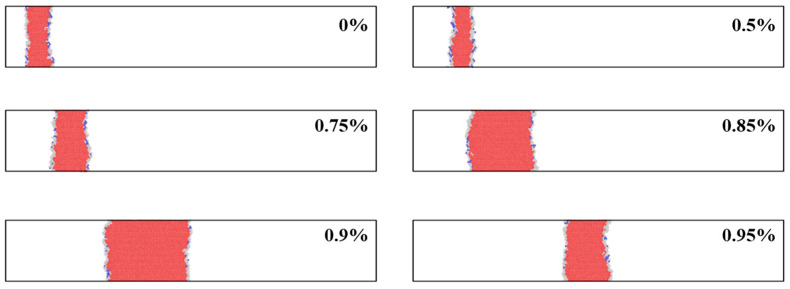
Evolution of the stacking-fault ribbon in the segregation-free and hydrogen-free model at different shear strains.

Figure 4 shows the evolution of the stacking-fault region in the segregation-free and hydrogen-free model at different shear strains. At the early stage of deformation, the dislocation moves only slightly on the slip plane, while the width of the stacking-fault ribbon gradually increases. With increasing strain, the stacking-fault region extends continuously along the slip direction, indicating the propagation of the two partial dislocations. When the strain reaches 0.95%, the stacking-fault width decreases slightly, suggesting that the trailing partial dislocation moves faster and reduces the separation distance between the two partials. This behavior is consistent with the typical glide of dissociated dislocations in FCC metals [23,24].

**Figure 5:**
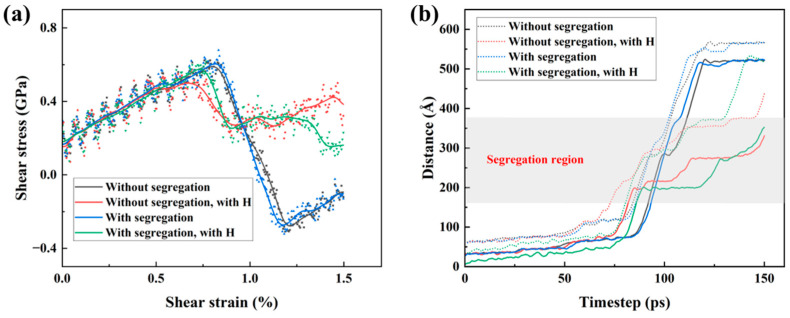
Effect of segregation and hydrogen on shear deformation behavior: (**a**) shear stress–strain curves of the different models; and (**b**) time evolution of the positions of the leading and trailing partial dislocations. The dashed lines and the solid lines in (**b**) represent the leading and trailing partial dislocation, respectively. The gray shaded area in (**b**) denotes the segregation region.

Following the structural characterization of dissociated dislocations, the effects of segregation and hydrogen on shear deformation were further analyzed from the shear stress–strain response and the evolution of dislocation positions, as shown in Figure 5. As shown in Figure 5a, all models exhibit a similar overall stress evolution, with the shear stress first increasing and then dropping after reaching the peak value, corresponding to the initiation and propagation of dislocations during deformation. Compared with the segregation-free model, the segregation-containing model without hydrogen does not show a significant change in the stress–strain response.

More detailed information is provided by the dislocation position–time curves in Figure 5b, where the dashed lines represent the leading partial dislocation and the solid lines represent the trailing partial dislocation. The gray shaded region denotes the segregation region. In the absence of hydrogen, segregation does not significantly change the propagation rate of the two partial dislocations or their separation distance.

**Figure 6:**
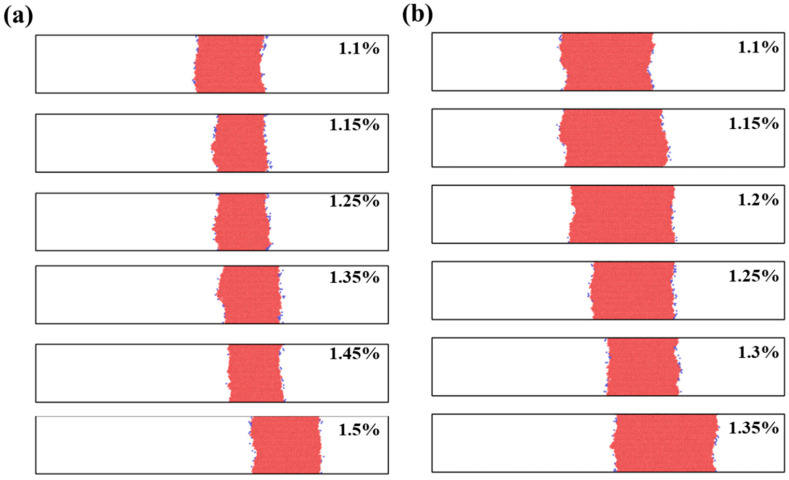
Snapshots of dislocation configurations in the two hydrogen-containing models at different shear strains during propagation across the segregation region. (**a**) without segregation, with H; (**b**) with segregation, with H.

By contrast, a more pronounced effect is observed in the hydrogen-containing models. When the dislocations pass through the segregation region, both the leading and trailing partial dislocations exhibit a longer residence time within this region. Further evidence is provided by the dislocation configurations in Figure 6 for the two hydrogen-containing models at different shear strains. In both cases, the leading partial dislocation shows a more obvious and prolonged residence near the segregation-region boundary before leaving the segregated zone. These results indicate that hydrogen enhances the retardation of dissociated dislocation motion in the segregation-containing region.

## 4. Discussion

The characterization of hydrogen in our models reveals a dual role in altering the material’s response. From an energetic perspective, hydrogen acts as a stabilizer for stacking faults by lowering the SFE, which promotes the dissociation of dislocations. From a kinetic perspective, the mobility of hydrogen is sensitive to local Ni/Cr concentrations, creating regions of varying hydrogen residency times. The coupling of these two effects manifests as the observed retardation of dislocation motion in the segregation zone. Specifically, the reduction in SFE increases the equilibrium width of the stacking-fault ribbon, while the local hydrogen environment provides an additional pinning-like resistance to the moving partials.

These findings indicate that local segregation affects hydrogen-related deformation in austenitic stainless steel mainly by modifying the local energetic and kinetic conditions for defect evolution, rather than by directly acting as a strong obstacle to dislocation motion. In the representative local-composition states considered here, the Ni-rich/Cr-lean region exhibits a higher stacking fault energy and faster hydrogen diffusion, whereas the Cr-rich/Ni-lean region shows a lower SFE and reduced hydrogen mobility. Hydrogen further decreases the SFE in all three alloy models, and its influence on local fault energetics is more pronounced than that of composition variation alone. Taken together, these results suggest that local segregation creates heterogeneity not only in defect energetics but also in hydrogen transport capability, thereby altering the local conditions under which hydrogen interacts with crystal defects [25,26,27,28,29,30,31].

This indirect role of segregation becomes clearer in the dislocation simulations. In the absence of hydrogen, the segregation band produces only a limited change in both the shear response and the propagation behavior of dissociated dislocations, indicating that local Cr/Ni redistribution alone has a relatively weak direct effect on dislocation motion in the present model. Once hydrogen is introduced, however, both partial dislocations remain in the segregation-containing region for a longer time, and the leading partial exhibits a more obvious delay near the segregation boundary. This behavior is qualitatively reminiscent of a local pile-up-like or temporary trapping effect at the segregation-region boundary [32,33], suggesting that the segregation zone, especially its boundary, may act as a transient trapping site for dislocation motion [34,35]. A reasonable interpretation is that the hydrogen-induced reduction in SFE increases the equilibrium separation of the two partial dislocations and weakens their coordinated glide, while the segregation-dependent hydrogen diffusivity affects the local redistribution of hydrogen during deformation [28,36,37]. Therefore, the observed retardation of dislocation motion should be understood as the combined consequence of local changes in fault energetics and hydrogen transport, rather than as a simple strengthening effect caused by composition variation alone.

These findings are relevant to SLM austenitic stainless steels, where rapid solidification commonly produces chemical heterogeneity at the cell, sub-grain, or boundary scale [3,15,16]. Crucially, our atomistic observations suggest that this segregation-hydrogen coupling has a detrimental effect on macroscopic mechanical properties. The H-induced reduction in stacking fault energy promotes localized planar slip, while the delayed dislocation motion within segregated zones leads to localized stress concentration. Consequently, segregation-containing regions in SLM microstructures act as preferential sites for crack initiation, leading to increased hydrogen embrittlement susceptibility and a loss of ductility under service conditions. The present results imply that reducing the severity of local segregation, perhaps through optimized processing parameters or post-heat treatment, may be beneficial for improving hydrogen tolerance in SLM-produced alloys.

Several limitations of the present study should be noted. The segregation states considered here are representative local-composition models rather than direct outputs of a full SLM solidification simulation, and the segregation morphology is simplified relative to real hierarchical microstructures. Additionally, the applied strain rate (1.0 × 10^−4^ ps^−1^) and the local hydrogen concentration (5 at.%) are higher than typical experimental conditions. However, the H-induced reduction in SFE is an intrinsic energetic property independent of the deformation rate, and the elevated H concentration represents the local enrichment of hydrogen often found at microstructural traps. We confirmed that no artificial phase transitions (e.g., hydride formation) occurred, ensuring that the observed retardation reflects the intrinsic influence of interstitial hydrogen on dislocation glide. Furthermore, the observation of dislocation retardation even at such high velocities indicates that segregation boundaries act as potent energetic barriers that would likely exert even greater influence at lower experimental rates where hydrogen-dislocation coupling is more fully realized. Therefore, the present results should be interpreted primarily as atomistic trends and mechanistic insights rather than direct quantitative predictions of macroscopic service behavior.

## 5. Conclusions

In this work, molecular dynamics simulations were performed to investigate the effects of representative local segregation on stacking fault energy, hydrogen diffusion, and dislocation motion in austenitic stainless steel. The results show that local composition variation significantly modifies both stacking fault energy and hydrogen diffusivity, with Ni-rich/Cr-lean regions exhibiting higher stacking fault energy and faster hydrogen diffusion, whereas Cr-rich/Ni-lean regions show lower stacking fault energy and reduced hydrogen mobility. Hydrogen further decreases the stacking fault energy in all three alloy models and exerts a stronger influence on local defect energetics than composition variation alone. In the shear simulations, elemental segregation by itself produces only a limited direct effect on dislocation propagation, whereas its interaction with hydrogen leads to a more evident retardation of partial dislocation motion within the segregation-containing region. Overall, the present results show that local segregation contributes to hydrogen-related deformation mainly by modifying local fault energetics and hydrogen transport, thereby providing an atomistic basis for understanding hydrogen-sensitive mechanical heterogeneity in SLM-relevant austenitic stainless steel.

## Data Availability

The original contributions presented in this study are included in the article. Further inquiries can be directed to the corresponding author.
